# Exploration of gene presence/absence variations in *Oncorhynchus mykiss* and their differentiation between wild and selection populations

**DOI:** 10.1098/rsob.240382

**Published:** 2025-05-21

**Authors:** Hancheng Bao, Na Xue, Boyuan Wang, Han Yu, Ming Huang, Jinghong He, Shuanglin Dong, Yangen Zhou, Qinfeng Gao, Yuan Tian

**Affiliations:** ^1^ Ocean University of China, Qingdao, Shandong, People’s Republic of China; ^2^ Auburn University, Auburn, AL, USA

**Keywords:** pan-genome, gene presence/absence variations, rainbow trout, artificial selection

## Abstract

Gene presence/absence variations (PAVs) have been considered as the important determinants of genome evolution and phenotypic diversity. However, studies on gene PAVs have been poorly documented, especially in fishes. In the present study, the pan-genome of rainbow trout was constructed based on 268 whole-genome re-sequencing accessions (4.38 Tb data). It recovered an additional 62 Mb sequences and 1288 protein-coding genes. Then, 9831 (22.77%) gene PAVs were genotyped across the 268 individuals. PAV-based PCA analysis, together with phylogenetic topology and STRUCTURE, revealed the clear separation among the different wild and selection populations. Additionally, a PAV-based genome-wide association study (GWAS) identified three candidate PAVs significantly associated with artificial selection. Meanwhile, fixation index analysis revealed 35 PAVs with significant frequency differences between wild and selection populations in Canada, while 15 candidate PAVs were detected between the populations in America. Their biological functions have been reported to participate in the regulation of growth performance and stress response. The present study deepens our understanding of widespread gene PAVs and facilitates the identification of key candidates that contribute to important traits.

**Figure:**
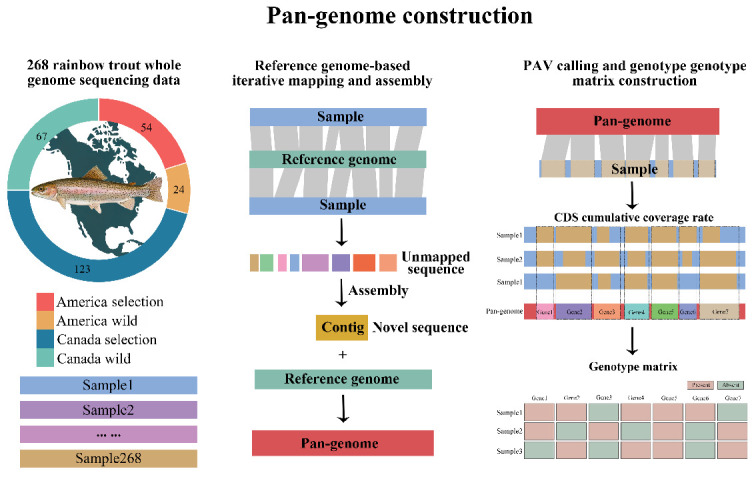

## 1. Introduction

Genetic variations that arise in the genome are proposed to be important sources of phenotypic diversity in evolution, although they are usually weakly deleterious and reduce fitness of organisms [1–3]. The amount and patterns of genetic variations are dramatically altered and shaped during long-term domestication and artificial selection breeding. Generally, genetic variations can be defined as a wide spectrum of variations of various sizes, ranging from single nucleotide polymorphism (SNPs) to much larger structural variants (SVs) [4,5]. So far, most studies only select SNPs to explore evolutionary mechanisms and genetic basis for improved economic traits, caused by long-term domestication and selective breeding. In sharp contrast, studies on the SVs have been largely neglected and poorly documented. Indeed, SVs harbour much larger phenotypic effects and act as ubiquitous drivers to phenotypic diversity in organisms, as they could influence at least 2–8 times more bases than SNPs [6,7]. Ongoing research has demonstrated that some SVs happen at the coding sequences and directly affect the completeness of functional genes, forming intra-specific variations in gene contents and defining gene presence/absence variations (PAVs) [8–10]. In fact, the concept of gene PAVs is originally noted in microbial and viral genomes, and subsequently unlocked in both plants and animals [10,11]. At present, gene PAVs have been considered as important determinants of genome evolution and phenotypic variability, inherently linked to local adaptation of wild population as well as some important agronomical traits, such as growth performance, abiotic and biotic stress tolerance in a range of species.

Owing to various gene PAVs among different individuals within the same species, a single reference genome hardly covers all the possible genetic information. Hence, the concept of pan-genome is proposed, representing the complete genome information and incorporating the gene sets of all or most individuals in one species. With the recent advances in high-throughput sequencing technology and bioinformatic tools, a large number of individuals' genomes have been sequenced, markedly facilitating their pan-genome construction and gene PAV genotyping. It provides a straightforward method for the detection of significant associations between gene PAVs and phenotypic traits. So far, an increasing number of pan-genome studies have been reported in plants and animals. There are two common approaches for pan-genome construction, including *de novo* assembly approach, and the iterative mapping and assembly approach [12,13]. The *de novo* assembly-based pan-genome generally depends on much long-read sequencing data are produced by PacBio or Nanopore technology [14–16]. In contrast, the pan-genome, based on the iterative mapping and assembly approach, could be achieved based on only a single reference genome and next-generation short-read sequencing data. Hence, the iterative assembly approach is often more practical and easier to implement in genetic research [13].

At present, the iterative mapping and assembly approach has been extensively applied in the pan-genome construction of many species, such as sorghum (*Sorghum bicolor*) [17], rice (*Oryza sativa*) [18], cotton (*Gossypium arboreum*) [19], human (*Homo sapiens*) [20], chicken (*Gallus gallus*) [21], pig (*Sus scrofa*) [10], goat (*Capra hircus*) [22], mussel (*Mytilus galloprovincialis*) [8] and Pacific oyster (*Crassostrea gigas*) [23]. Moreover, this approach could identify the gene PAVs in more individuals and capture more important genetic information. For example, the pan-genome of sorghum reveals substantial gene content variations with approximately 64% of gene families PAVs. Of them, variation in the SbRc gene has proven to be significantly associated with grain-colour parameters in sorghum [17]. Based on the 250 sequenced individuals from 32 breeds in Eurasia, the pan-genome of pigs was constructed to generate 308.3 Mb non-reference sequences and 3438 novel genes absent in the current reference genome. Additionally, a substantial proportion of protein-coding genes (16.8%, 4135) are variable among these pigs, which have been undergone stronger selective pressures with less functional conservation during artificial selective breeding. Gene PAVs in chickens are observed to be shaped by artificial selection, genetic drift and hybridization. More importantly, it was found that the gene content of IGF2BP1 is a causal variant for body sizes in chickens, providing a useful resource for biological discovery and breeding [21]. In mussels, there are approximately 38% protein-coding genes with PAVs among 234 accessions that are found to be enriched in immune and stress-response functions and tightly linked to the local adaptation to different marine coastal environments among populations [8]. These well-documented cases provide strong evidence for the remarkable phenotypic effects of gene PAVs. However, the full spectrum of gene PAVs has not been systematically investigated in most organisms.

Rainbow trout (*Oncorhynchus mykiss*) are typically cold-water fish with native ranges extending from California in the eastern Pacific to the Kamchatkan Peninsula in eastern Siberia [24]. In 1870s, the California Acclimatization Society conducted the first domestication of rainbow trout by artificial propagation of wild individuals [25]. Then, long-term artificial selective breeding was initiated for the genetic improvement of economic traits, generating excellent growth performance, stronger temperature tolerance and resistance to disease [26,27]. Hence, rainbow trout has been introduced into at least 80 countries with abundant cold-temperature water resources and established reproducing populations, becoming one of the most widely farmed freshwater fish around the world [28]. Given the economic importance, rainbow trout is undoubtedly considered as important model species for genetic study. In the last decade, numerous studies have been conducted to explore and uncover the genetic basis of important agronomical traits in rainbow trout. For instance, a total of 247 SNPs are reported to be significantly associated with body weight gain in rainbow trout, and the SNP-harbouring genes are involved in cell growth, cell proliferation and lipid metabolism [29]. GWAS revealed that SNPs located in Omy27, Omy17 and Omy9 could be related to fillet yield of rainbow trout, explaining approximately 4.4% genetic variances [30]. There are 10 QTLs for IHNV resistance identified by genome-wide scan of SNP frequency with moderate effects and many loci with small effects [31]. In addition, immune resistance to bacterial cold-water disease [32,33], *Flavobacterium columnare* [34] and *Yersinia ruckeri* infection [35] also received much attention. However, these genetic documents are only focused on the single-nucleotide variations, disregarding the potential roles of SV and the associated gene PAVs in phenotypic variations.

In the present study, an iterative assembly strategy was used to construct the pan-genome of rainbow trout based on wild and artificial breeding populations consisting of 268 accessions. Then, gene PAVs were accurately genotyped to explore the genetic structure and diversity among individuals. Additionally, these high-quality PAVs were also used for the GWAS and selective signal analysis for the identification of candidates that significantly associated with long-term artificial breeding. The present study would deepen our understanding of genetic diversity and facilitate the identification of key genetic variations that contribute to important traits. Moreover, it would offer new approaches and insights for studying genetic basis of fish breeding, informing the development of selective breeding strategies to enhance the sustainability and productivity of aquaculture fisheries.

## 2. Results

### 2.1. Pan-genome construction of rainbow trout

**Figure 1.**
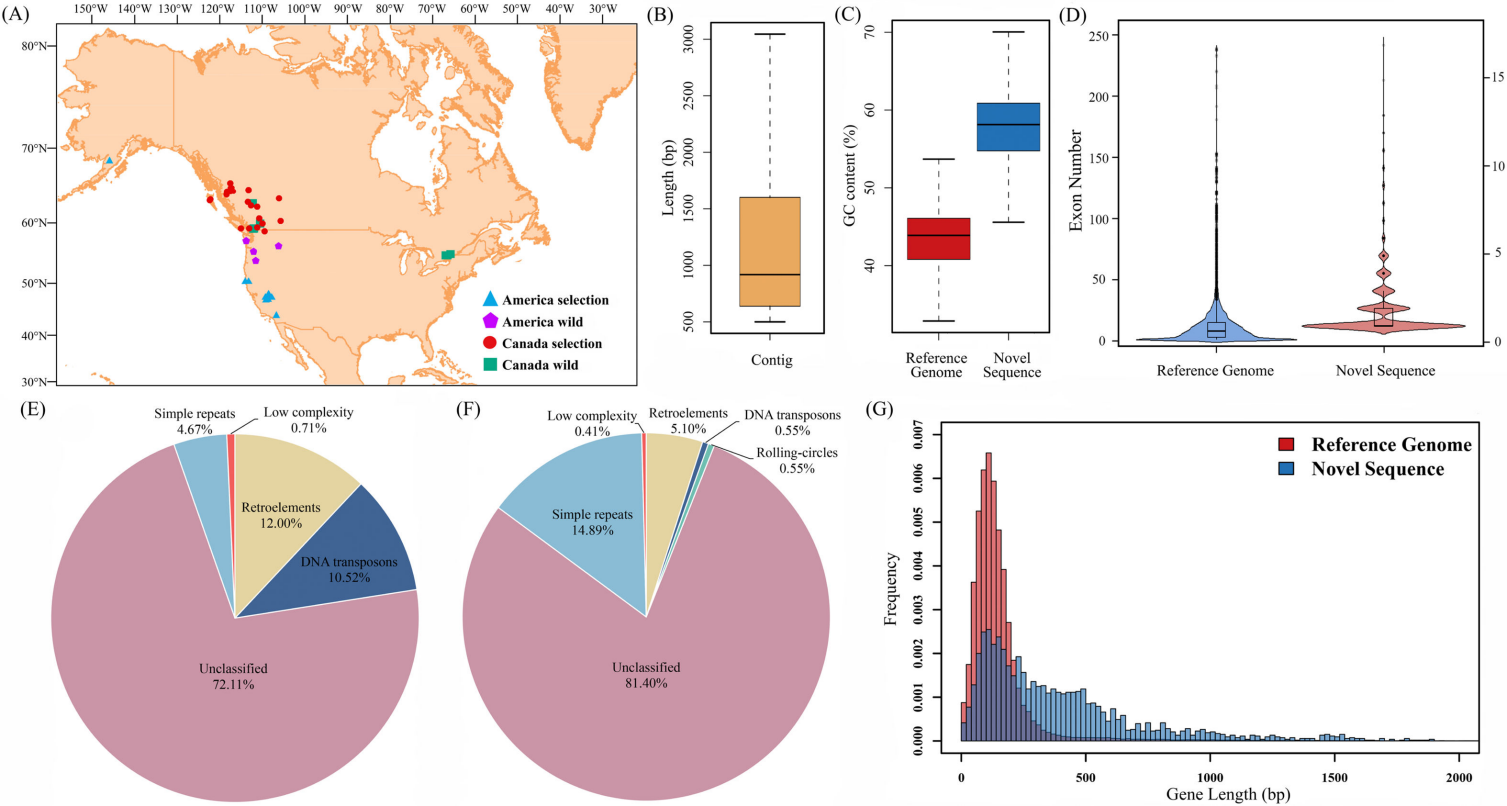
Pan-genome of rainbow trout. (A) Geographical distribution of rainbow trout accessions used for the construction of pan-genome. (B) Distribution of novel contig lengths. (C) GC contents of novel sequences and reference genome. (D) Exon numbers distribution of the reference genome and novel sequences; (E) Repeat sequences in the reference genome. (F) Repeat sequences in the novel sequences. (G) Frequency of protein-coding gene lengths in the reference genome and novel sequences.

In the present study, the pan-genome of rainbow trout was constructed by the iterative mapping and assembly approach based on the OmykA_1.1 genome and 268 whole-genome re-sequencing (WGS) accessions, containing 4.38 Tb WGS data (figure 1A). These rainbow trout were mainly distributed in the Pacific Northwest regions of America (78) and Canada (190) (figure 1A). De novo assembly of these unmapped short reads produced 39 073 contigs, longer than 500 bp (figure 1B). While the assembled contigs were found to be largely varied in length. The sizes of contig N50 was estimated at 2130 bp, while the largest contig was able to reach 576 560 bp. In total, all the assembled contigs comprised 62.06 Mb novel sequences, absent in the reference genome. Additionally, it was found that the GC contents of novel sequences (56.24%) were much higher than the reference genome (42.89%) (figure 1C). This result could be attributed to the complex sequence features with the extreme GC-content variation in gap regions of the reference genome.

The percentages of identified repeat sequences varied dramatically between novel sequences (approx. 7.26%) and the reference genome (approx. 55.25%) (figure 1E,F), probably due to the limitation of de novo assembly by short reads. Despite the differences in the proportion of repeat sequences, most of them remain unclassified in both novel sequences and reference genomes. However, it was found that the patterns of known types were largely different, such as abundant retroelements in the reference genomes and rich simple repeats in novel sequences. Protein-coding gene prediction analysis revealed 1288 high-confidence genes (AED ≤ 0.2) in the novel sequences, the lengths of which were observed to be similar to those in the reference genomes (figure 1G). However, there also existed some relatively longer genes with many more exons in the novel sequences. In order to verify the reliability and accuracy of protein-coding genes in novel sequences, 128 RNA-seq datasets, generated by our research group or published in the SRA database, were used for expression analysis. As a result, 981 genes were found to be expressed among these individuals, despite the different abundance. It strongly supported the reliability of predicted protein-coding genes in novel sequences (electronic supplementary material, figure S1). Finally, the pan-genome of rainbow trout was formed by the combination of novel sequences and reference genomes, resulting in 2403 Mb sequences and 43 171 protein-coding genes.

### 2.2. Gene content and presence/absence variation analysis

**Figure 2.**
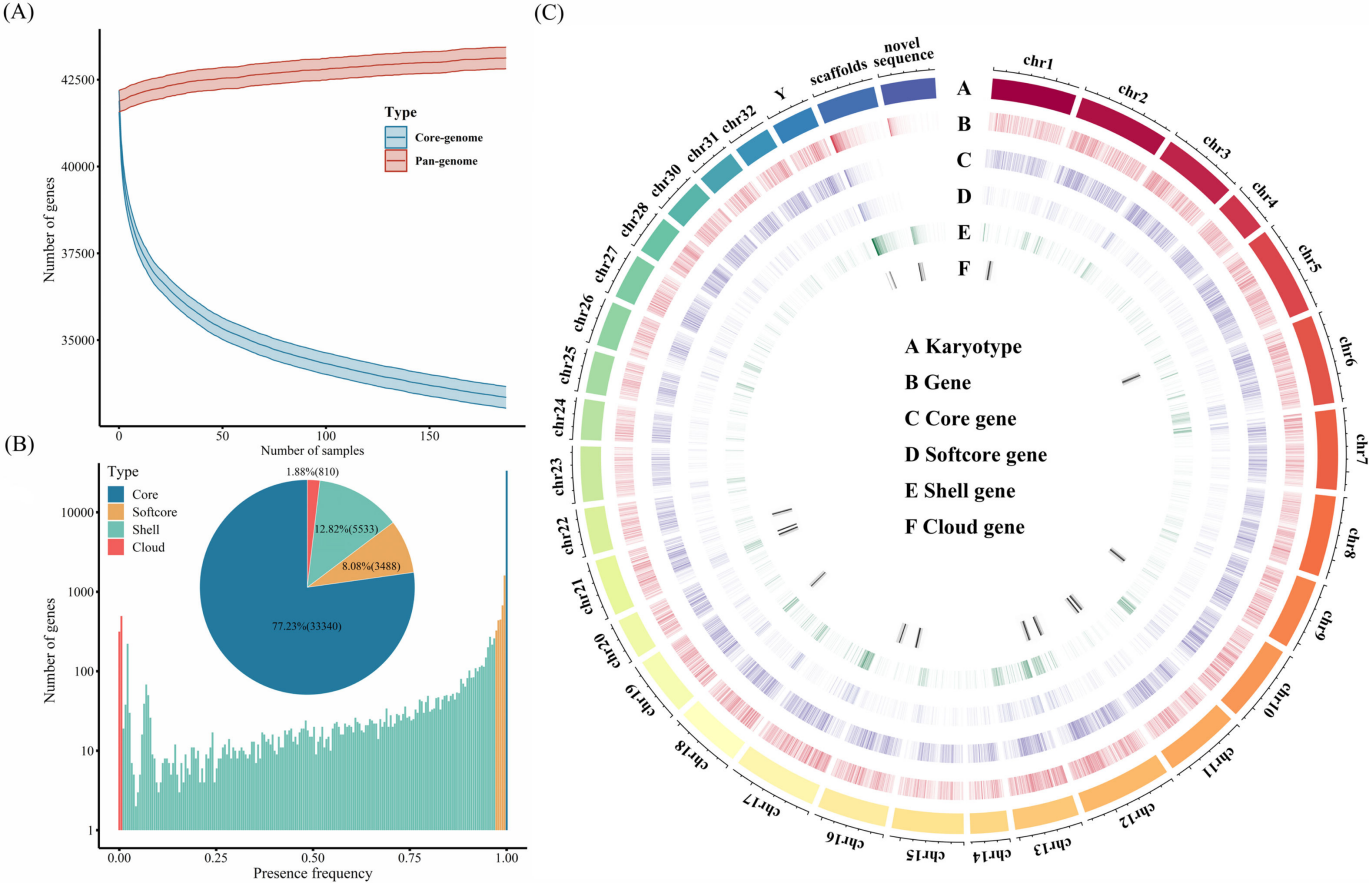
Gene content and PAV analysis in 268 rainbow trout accessions. (A) Variations of core and variable genes in the pan-genome with additional rainbow trout accessions. (B) Frequency of core, softcore, shell and cloud genes in the pan-genome of rainbow trout. (C) Genome-wide distribution of core, softcore, shell and cloud genes.

According to the frequency variations of presence/absence, the protein-coding genes in the pan-genome of rainbow trout were first categorized in core and variable types. As adding the additional rainbow trout accessions, the number of core genes decreased dramatically and approached a plateau when *n* = 170 (figure 2A). In sharp contrast, the number of variable genes was paralleled to rainbow trout accessions (figure 2B). In total, most genes 33 340 (77.23%) belonging to core genes were consistently present in 268 rainbow trout. The remaining genes (9831, 22.77%) were variable with the presence and absence variations in these accessions, which would be further classified into three types, including softcore, shell and cloud. Specially, 5533 genes presented in 260–268 accessions (>97%) were defined as softcore genes, 28 679 genes presented in 3–259 accessions (1–97%) were defined as shell genes, and 27 genes presented in one to two accessions were defined as cloud genes (figure 2B). As a result, each accession had core genes that varied between 80.26 and 86.70% and at most 19.74% of variable genes. It suggested the variability and complexity of gene contents in genomes among rainbow trout individuals. Additionally, the genome distribution of core and variable genes was further explored in the present study (figure 2C). It was found that core genes were mainly located in the centric regions of chromosomes, while the variable genes, especially softcore and shell types, preferred to concentrate at the ends of chromosomes, such as N-terminal regions of chr1, chr10, chr13 and C-terminal regions of chr5, chr12 and chr15. It indicated that the stability and communication of DNA structures and gene contents in the centric regions of chromosomes instead of terminal regions.

**Figure 3.**
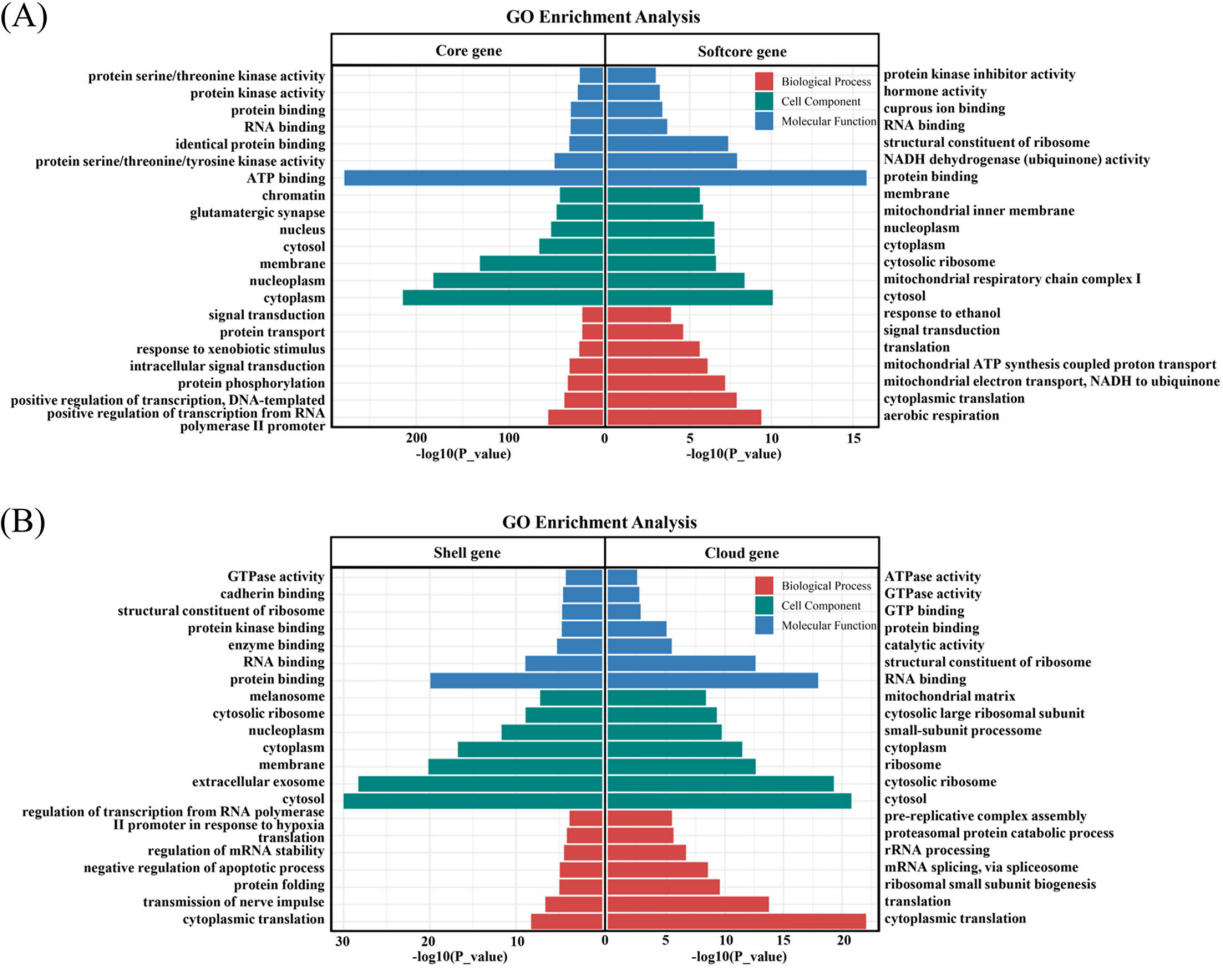
GO enrichment analysis of various gene types. (A) Enriched GO terms of core and softcore genes. (B) Enriched GO terms of shell and cloud genes.

In order to investigate the biological functions of various gene types, GO enrichment analyses were performed for the core genes, softcore genes, shell genes and cloud genes, respectively (figure 3). The results indicated that these core genes were significantly enriched in some GO terms associated with protein binding, ATP binding and identical protein binding (figure 3A). These biological functions seem to be crucial for the survival, growth, development and reproduction of organisms. Softcore genes are mainly involved in aerobic respiration and ATP synthesis. In addition, cytoplasmic translation, signal transduction and negative regulation of the apoptotic process were identified in the enriched GO terms of shell and cloud genes. There appears to be gene content diversity and functional divergence in response to biotic and abiotic stresses among these rainbow trout individuals.

### 2.3. Presence/absence variation-based population structure analysis

**Figure 4.**
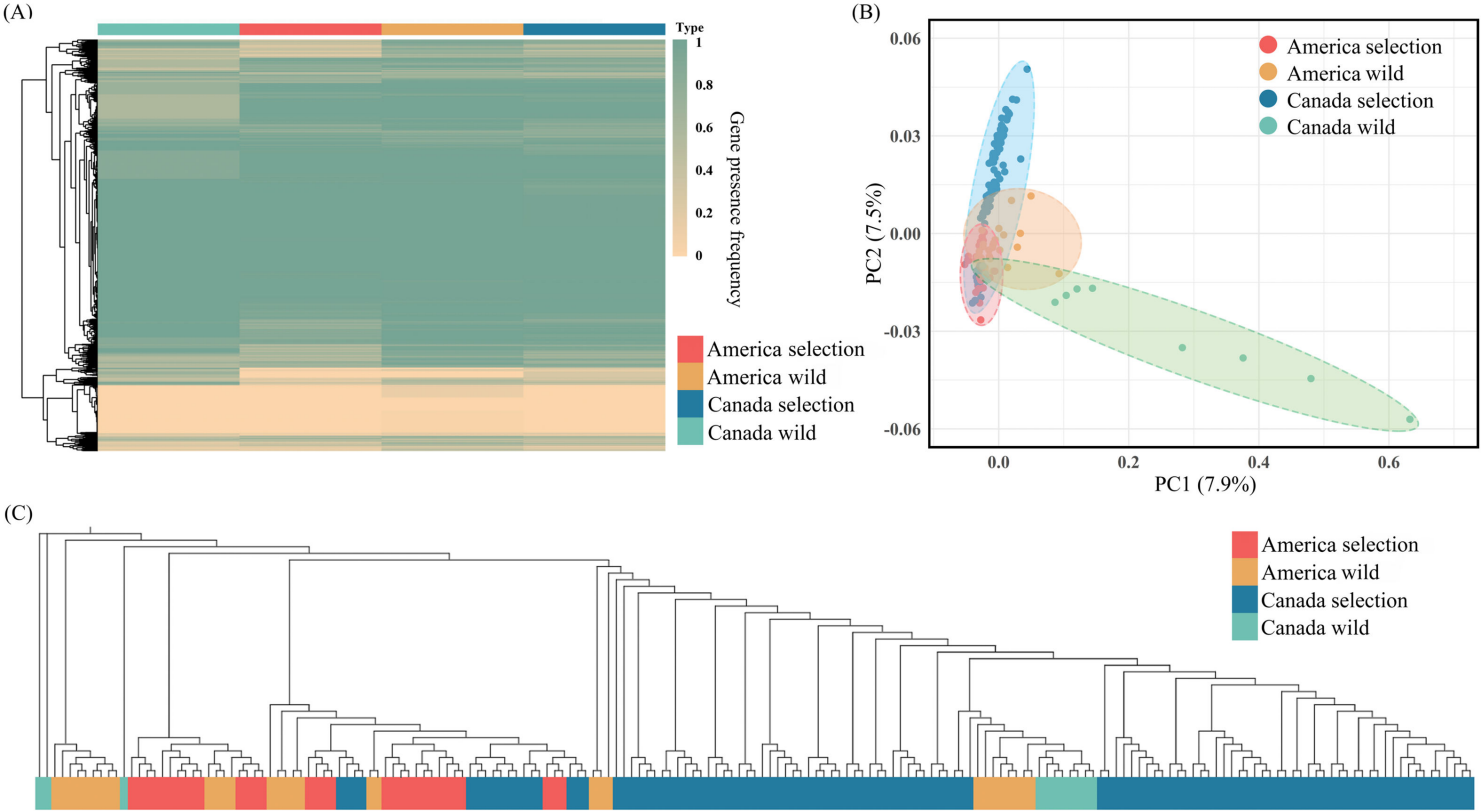
PAV-based genetic structure analysis. (A) Gene contents and PAV distribution within wild and selection populations of rainbow trout. (B) PAV-based PCA. (C) Phylogenetic tree of rainbow trout from wild and selection populations. The tree was constructed based on gene PAV information by the neighbour-joining method.

Gene contents and their PAV distribution were largely different between wild and selection populations of rainbow trout, representing the substantial variations of genetic structures affected by long-term artificial selection (figure 4A). Wild populations of rainbow trout, from either America or Canada, were found to encompass more variable genes relative to the selection population (figure 4A). It was consistent with the great genetic diversity of wild populations. Principal component analysis (PCA) and phylogenetic analysis were conducted to further investigate the genetic structures of wild and selection populations based on their gene contents and PAV information (figure 4B,C). The PCA results revealed that rainbow trout from different populations were obviously separated into distinct clusters, suggesting their differences in the genetic structure. Individuals from the wild populations, especially Canada’s wild populations, displayed larger genetic distances with only a few exceptions. It could provide additional evidence for the great genetic diversity of wild populations. In contrast, rainbow trout from selection populations were clustered more tightly, which may be partially attributed to the intense selection, hindrance of gene flows and introgression during artificial selection. Additionally, strong correlation was found to be existed between phylogenetic topology and principal component, confirming the reliability and accuracy of genetic structure analysis based on the PAV information (figure 4C).

### 2.4. Presence/absence variation-based genome-wide association study for artificial selection in rainbow trout

**Figure 5.**
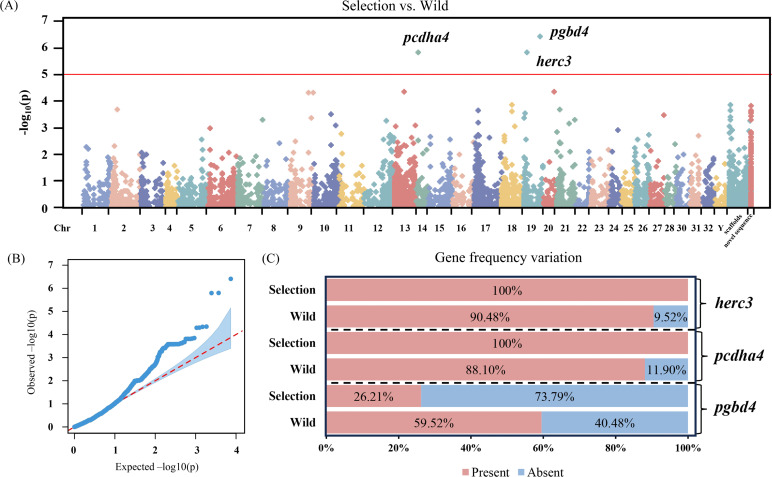
PAV-based GWAS for gene contents significantly associated with artificial selection. (A) Manhattan plot shows significant PAVs and gene contents associated with artificial selection. (B) Q–Q plot (quantile–quantile plot) showed gene occurrence frequencies in the selection/wild traits. Blue dots in QQ plots represent the observed –log10(*p*) values for the study, and the red dotted line denotes the expected –log10(*p*) values for the study. (C) Presence or absence frequency of significant PAVs in wild and selection populations.

According to the gene content and PAV information of 91 rainbow trout from the wild population and 177 individuals from the selection population, GWAS was conducted for the detection of significant PAVs associated with artificial selection (figure 5A).

A total of four common models for association analysis (GLM, MLM, MMLM and FarmCPU) were tested and compared according to the consideration of the false positive in trait-marker associations (electronic supplementary material, figure S2). As shown in the quantile–quantile (Q–Q) plot, GLM, MMLM and FarmCPU models were obviously deviated from expectation. The MLM model fitted well to the association analysis. As a result, the frequencies of three genes, including *herc3* (*HECT and RLD domain-containing E3 ubiquitin protein ligase 3*), *pcdha4* (*protocadherin alpha−4*) and *pgbd4* (*piggybac transposable element derived 4*), were found to reach the significant threshold for the association with artificial selection. The contents of significant PAVs were further investigated in wild and selection populations. All the rainbow trout from the selection population harboured the complete *herc3* and *pcdha4* genes, relative to approximately 10% absence in those of the wild population (figure 5C). It was noted that the presence frequency of the *pgbd4* gene, as a possible unfavourable gene, was dramatically decreased from 59.52% in the wild population to 26.21% in the selection population. The genetic effects of the *pgbd4*, *herc3* and *pcdha4* genes were estimated as 0.036, 0.067 and 0.0622, respectively. The retention or loss of functional genes could be random or due to respective positive or negative selection.

### 2.5. Presence/absence variation-based selection signal analysis for artificial selection in rainbow trout

**Figure 6.**
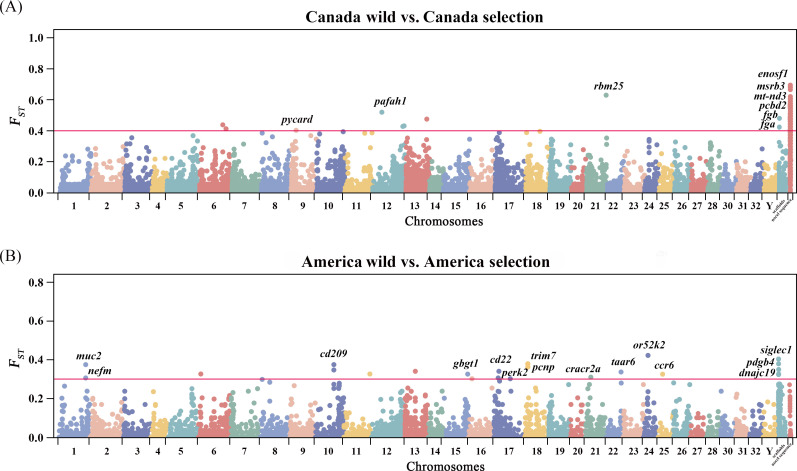
Genome-wide distribution of PAV-based selective sweeps identified by *F*_*ST*_ in rainbow trout. (A) *F*_*ST*_ analysis for PAV-based selective sweeps between Canada wild and selection populations. The horizontal red line represented the top 1% threshold in *F*_*ST*_ value (0.40). (B) *F*_*ST*_ analysis for PAV-based selective sweeps between the America wild and selection populations. The horizontal red line represented the top 1% threshold in *F*_*ST*_ value (0.33).

To identify the selection signals of gene PAVs during artificial selection in rainbow trout, fixation index (*F*_*ST*_)analysis was utilized for the two sets of comparisons of flexible gene frequencies, between wild and selection populations in Canada (figure 6A), and wild and selection populations in America (figure 6B). For each comparison, genes with top 1% frequency differences were considered as significant candidates for the selection signals. In total, the frequencies of 35 gene PAVs significantly differed between wild and selection populations from Canada, which were regarded as selection signals from the artificial selection (figure 6A). These candidates of gene PAVs were unevenly distributed across the chromosomes of rainbow trout, and 26 members in novel sequences attracted much attention. Meanwhile, *F*_*ST*_ analysis revealed 15 gene PAVs with significant differentiation of frequencies between wild and selection populations from America (figure 6B), appeared in 33 auto-chromosomes of rainbow trout.

**Figure 7.**
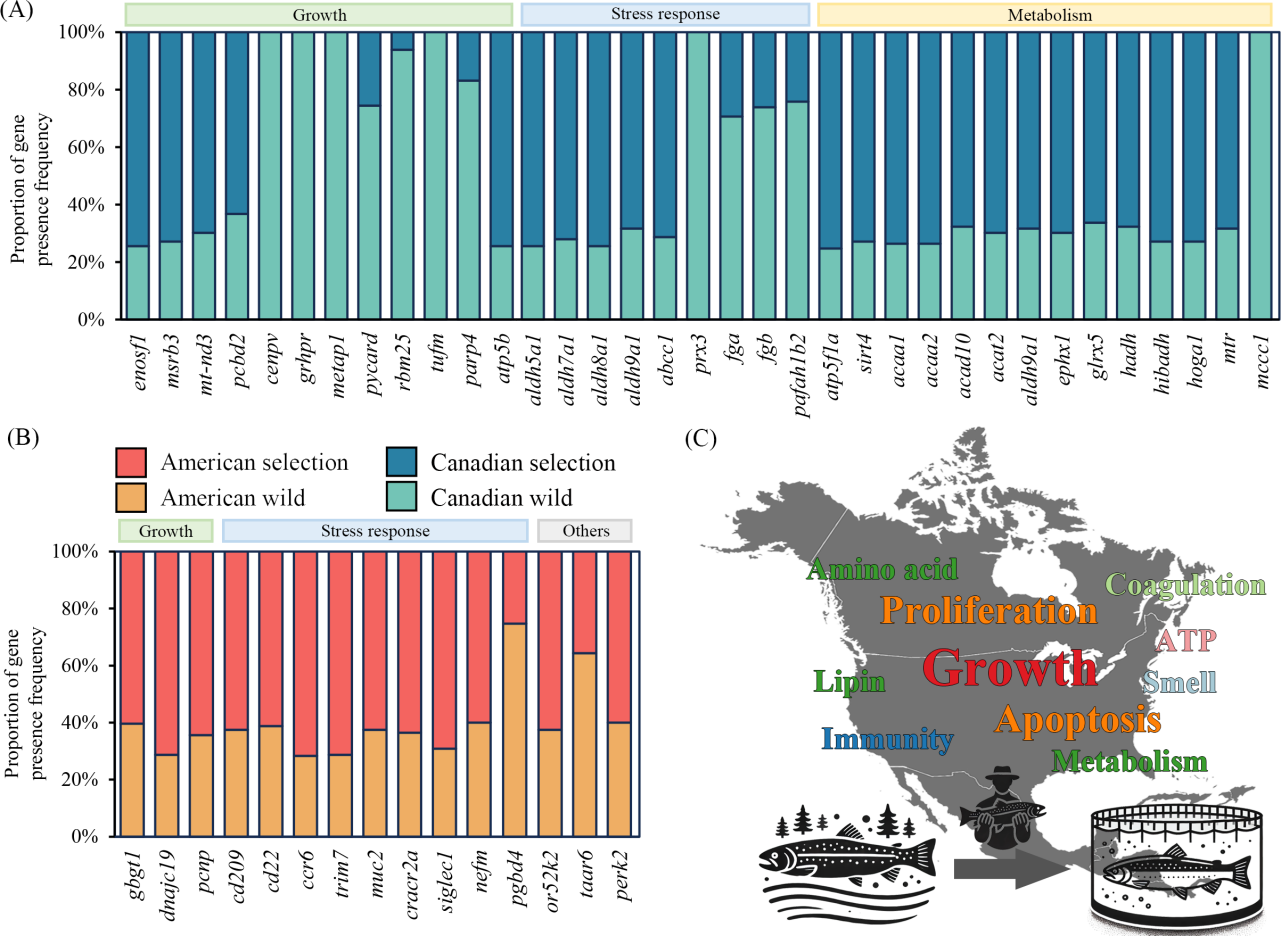
Frequency statistics of candidate gene PAVs. (A,B) Proportions of candidate gene PAVs in rainbow trout population of Canada and America. (C) Word cloud showing the potential biology functions of candidate gene PAVs. The font size represents the frequency of occurrence.

The changes in the frequency of candidate gene PAVs were systematically investigated and summarized to better understand the selection signatures between wild and selection populations of rainbow trout. More than half of the candidates (22, 62.9%) exhibited increased frequencies in selection population relative to wild population in Canada. Of these candidates, 11 genes (*enosf1*, *msrb3*, *mt-nd3*, *pcbd2*, *cenpv*, *grhpr*, *metap1*, *pycard*, *rbm25*, *tufm*, *parp4*) were closely associated with cell growth and proliferation, nine genes (*aldh5a1*, *aldh7a1*, *aldh8a1*, *aldh9a1*, *abcc1*, *prx3*, *fga*, *fgb*, *pafah1b2*) were believed to be involved in stress response, 15 genes (*atp5b*, *atp5f1a*, *sirt4*, *acaa1*, *acaa2*, *acad10*, *acat2*, *aldh9a1*, *ephx1*, *glrx5*, *hadh*, *hibadh*, *hoga1*, *mtr*, *mccc1*) were closely related to energy metabolism, amino acid metabolism and lipid metabolism (figure 7A).

Additionally, we also found that the frequency of 15 candidate genes were significantly altered and improved in the selection population of America. These genes with increased frequencies could be categorized into growth, stress response, olfactory sensation and circadian rhythms. For example, *gbgt1*, *dnajc19* and *pcnp* genes worked for regulation of growth performance, *cd209*, *cd22*, *ccr6*, *trim7*, *muc2*, *cracr2a*, *siglec1*, *nefm* and *pgbd4* genes participated in stress response. Moreover, *52 k2,* and *taar6* genes were related to olfactory sensation, *perk2* gene was related to circadian rhythms (figure 7B). A conserved selection preference from both Canadian and American populations of rainbow trout was observed for growth performance and stress response, which seem to be important traits in artificial selection breeding of either economic plants or animals (figure 7C).

## 3. Discussion

### 3.1. Pan-genome construction

Pan-genome generally represents the entire genomic repertoire and DNA diversity of a species. It is expected to have better accuracy and completeness than the commonly used single linear reference genome, allowing us to further explore the variations and elements within genomes [36,37]. In the present study, the pan-genome of rainbow trout was first constructed based on the linear reference genome and 268 WGS datasets by the method of iterative assembly mapping strategy. It recovered additional 62 Mb sequences and 1288 protein-coding genes for the reference genome of rainbow trout. Similarly, a series of novel sequences are also captured in chickens (66.5 Mb) [21], ducks (33 Mb) [38], pigs (72.5 Mb) [39] and humans (276 Mb) [40]. We have systematically investigated the sequence characteristics of the reference genome and novel sequences in rainbow trout. Obviously, there were relatively higher GC contents of novel sequences, rather than the reference genome. It is paralleled to the characteristics of novel sequences in chickens [41], ducks [42], pigs [10] and humans [40]. As known, GC bias usually hinders genome assembly and negatively affect the completeness, especially the short-reads-based assembly [43]. GC bias may be responsible for the assembly error or absence of these novel sequences in the reference genome. The differences of repeat sequence types could be attributed to the lengths of sequences, such as the rich simple repeats in novel sequences.

In the last decade, gene content and PAVs have been extensively studied in bacterial and viral genomes, due to their small sizes, simple organization and fast gene gain, loss and horizontal transfer rates [44,45]. Recently, with the development of high-throughput sequencing and advances in eukaryotic pan-genome studies, gene content and PAVs are occasionally reported in plants and animals, such as sorghum [46], soybean [47], chicken [21] and Mediterranean mussel [8]. The intra-species gene content and PAVs may be attributed to genetic drift, hybridization, domestication and artificial selection [21]. Under the guidance of the pan-genome, we have revealed the gene content and PAVs across the 268 rainbow trout accessions, resulting in approximately three-fourths of core genes and three-fourths of variable genes. A large majority of protein-coding genes, belonging to core genes, were present across all the rainbow trout with high stability, which may be fundamental for survival. These variable genes, selectively present or absent in a subset of the individuals, are usually believed to participate in accessory functions. It was found that 71.54% of core genes in rainbow trout were tightly to related to protein binding. Protein binding is fundamental to life, as protein–protein interactions are essential for processes like signal transduction, enzymatic activity, transcriptional regulation, cytoskeletal organization and molecular transport. Protein-binding-related genes in the core genes encode critical components such as signalling molecules, receptors, transcription factors and structural proteins. These genes are highly conserved due to their indispensable roles in survival and centrality within complex biological networks. Core genes also exhibit broad functionality, supporting genome stability, transcriptional and translational regulation and metabolic networks through protein binding. Additionally, the modularity and flexibility of protein-binding functions enable these genes to participate in multiple biological pathways and adapt to various conditions. As central nodes in molecular networks, the loss of such genes can disrupt entire systems, underscoring their critical role as core genes.

### 3.2. Artificial selection for gene contents and presence/absence variations

The genetic structure and diversity of animals are substantially altered by long-term domestication or artificial selection [48,49]. Detection of SNPs and SVs generally works to identify selection signature and discover the genetic basis between wild and selected populations. In the present study, it was found that artificial selection also caused the genetic differences in gene contents among different populations of rainbow trout. This result could be strongly supported by the considerable divergences in PAV-based genetic structure. These variable genes, between wild and selected populations, are believed to evolve rapidly and contribute significantly to the phenotypic variations of economic traits in rainbow trout, such as improved growth performance and enhanced resistance to disease infection.

To infer the selection signal and determine the significant association between gene contents and artificial selection, PAV-based *F*_*ST*_ analysis, together with GWAS, was performed for these variable genes in wild and selection populations of rainbow trout from both Canada and America. The results revealed a series of candidate genes, the contents or frequencies of which underwent divergences between wild and selection populations. It clearly points out the fraction of protein-coding genes that heavily affected by artificial selection of rainbow trout. Additionally, significant associations between gene contents and artificial selection have also been reported in the previous documents of chicken [21] and Mediterranean mussel [8]. Changes in gene contents or frequencies represent a major motif of molecular evolution and a common evolutionary response of populations undergoing a shift in environment and, a change in the pattern of selective pressures [50,51]. It proposed to be an important source of phenotypic diversity and environmental adaptation in evolution.

### 3.3. Candidate presence/absence variation of gene contents for economic traits

Investigation of candidate genes is of significance to understanding their phenotype effects on rainbow trout during artificial selection breeding. It was noted that PAV-based GWAS have identified three gene PAVs with significant association to the breeding process, including *pcdha4*, *herc3* and *pgbd4*. Their biological functions have been investigated as follows: as a member of the *protocadherin alpha* gene family, *pcdha4* is typically involved in the development of the neural system [52,53]. It has been well proven that there are divergent presence/absence frequencies of *pcdha4* among distinct chicken populations in China, which could regulate the egg laying production by affecting the development of neural system [54]. Hence, it is believed that increased frequencies of *pcdha4* may be responsible for the excellent egg production performance of farm-cultured rainbow trout. The protein encoded by *herc3* gene, has the ability to inhibit viral replication by the interacton with *interferon-stimulated 15* (*isg15*) gene and promote the ubiquitylation of viral proteins [55,56]. In the macrophage-like cells of Atlantic salmon, expression of *herc3* is significantly induced when confronted with viral mimic pIC stimulation [57]. Therefore, increased *herc3* genes in rainbow trout underwent artificial selection for stronger defence against virus. The *piggyBac* system is generally accepted as a typical Class II transposable element that harbours high transposition activity with the ability to affect the sequence composition of genomes [58]. Indeed, it has been reported that *piggyBac* is dramatically decayed in most mammalian genomes caused by structural variations, which would cease their transposition and reduce genome shaping during the later phase of primate radiation [59,60]. This striking finding is in agreement with the observation in the present study. Increased absence of *piggyBac* in the selection population of rainbow trout may be associated with improvement in genome stabilization after artificial selection.

In addition, wild and selection rainbow trout from Canada and America were obviously separated for the independent *F*_*ST*_ analysis to detect the gene PAV differentiation and selective signatures. As resulted, 50 gene PAVs with notable allele frequency differences were identified as the important candidates. Although there were different patterns of selection signatures between the rainbow trout populations from Canada and America, these candidates could be commonly related to growth performance and stress response.

In rainbow trout of Canada, artificial selection breeding enhanced the presence frequencies of *enosf1*, *msrb3*, *mt-nd3* and *pcbd2*, while resulted in the absence of *cenpv*, *grhpr*, *metap1*, *pycard*, *rbm25*, *tufm* and *parp4*. Emerging evidence has revealed that *enosf1*, *msrb3*, *mt-nd3* and *pcbd2* are positive to cell proliferation and differentiation, participating in the growth and development of organisms [61–63]. However, *grhpr*, *metap2, pycard*, *rbm25* and *tufm* [64–67] would negatively affect the cell cycles and inhibit protein synthesis [68,69]. Reduction of presence frequencies may contribute to the growth performance in selection population of rainbow trout in Canada. In addition, members of the aldehyde dehydrogenase gene family (*aldh5a1*, *aldh7a1*, *aldh8a1* and *aldh9a1*) were found to be impacted and showed divergent presence frequencies between the wild and selection populations. Increased aldehyde dehydrogenase would mitigate oxidative/electrophilic stress and improve the stress tolerance of rainbow trout [70,71]. There existed the relatively high presence frequencies of functional genes associated with amino acid (*hibadh, hoga1, mtr, mccc1*), lipid (*hibadh, hoga1, mtr, mccc1*) and ATP (*atp5b, atp5f1a, sirt4*) metabolism. Obviously, artificial selection effectively altered the metabolic capacity of rainbow trout, making it more adaptable and productive in the aquaculture industry.

In addition, *F*_*ST*_ analysis revealed three more candidate genes related to growth regulation in rainbow trout from America, including *gbgt1*, *pcnp* and *dnajc19*. Of them, *gbgt1* is able to regulate the glycosphingolipid biosynthesis [72]. More importantly, it is regarded as an important candidate QTL gene for the growth performance in small yellow croaker (*Larimichthys polyactis*) [73]. Both *dnajc19*- and *pcnp*-knockdown markedly hinder cell growth by mediating the PI3K-AKT signalling pathway [74,75]. Compared with wild population, their presence frequencies were enhanced in selection population that could promote the growth of rainbow trout. We have also found that candidate genes, including *cd209*, *cd22*, *ccr6*, *trim7*, *muc2*, *cracr2a*, *siglec1* and *nefm*, were involved in stress response, especially immunity against pathogen infection [76,77]. Accumulating documents provide strong evidence for their important roles in innate and adaptive immunity in numerous teleosts, such as half-smooth tongue sole (*Cynoglossus semilaevis*), rainbow trout [78,79], turbot (*Scophthalmus maximus*) and blunt snout bream (*Megalobrama amblycephala*), grouper (*Epinephelus coioides*) [80,81]. In the present study, rainbow trout that underwent long-term artificial selection breeding harboured the higher presence frequencies of immune-related genes compared with those in wild populations, which could make great contributions to the improvement of immunity and stress response.

## 4. Conclusion

In the present study, we have constructed a pan-genome of rainbow trout by the method of iterative assembly mapping strategy, recovering an additional 62 Mb sequences and 1288 protein-coding genes with abundant expression levels. Gene PAVs were fully genotyped across the 268 rainbow trout individuals, according to their frequency variations of presence/absence. Functional analysis suggested that core genes, present in all the individuals, were fundamental for survival, while genes with PAVs were tightly linked to various phenotypic traits. PAV-based PCA analysis, paralleling with STRUCTURE and phylogenetic tree, revealed the clear separation and considerable divergences in genetic structure among distinct rainbow trout populations. It reflected the diversity of gene PAVs and uncovered the complexity of genomic architectures in rainbow trout. Moreover, a series of gene PAVs were identified as important candidates with significant association or strong selection signatures to long-term artificial selection breeding. All the candidate genes, from either Canada or America rainbow trout populations, have been reported to participate in the regulation of diverse agronomic traits, especially growth performance and stress response. The present study provided valuable insights for diversity and complexity of widespread gene PAVs in rainbow trout, and formed a basis to further elucidate the genetic mechanisms and phenotypic effects of large-scale gene PAVs on agronomic traits in fishes.

## 5. Method details

### 5.1. Sample and data collection

In the present study, a total of 268 rainbow trout, widely distributed in America (78) and Canada (190), were selected to construct the pan-genome and detect the variations of gene contents. Of them, 91 individuals were wild and the others (177) were derived from artificial selection. The whole-genome resequencing (WGS) datasets of 268 accessions were obtained and prepared from the public Sequence Read Archive (SRA) database with the BioProject ID of PRJNA386519, PRJNA803495 and PRJNA402066. These WGS data consisted of 150bp pair-end reads, generated by the Illumina sequencing platform. A complete list of the accessions used in the present study was provided in electronic supplementary material, table S1.

### 5.2. Pan-genome construction

The pan-genome of rainbow trout was constructed by the reference-based iterative mapping and assembly approach. The public OmykA_1.1 assembly worked as a starting reference genome. This approach allowed the use of the WGS datasets of many individuals with genetic diversity to construct a pan-genome. In brief, raw reads from each accession were processed to filter out low-quality reads and generate clean reads using fastp (v. 0.23.2) software with default parameters. FASTQC (v. 0.12.1) software was used to check and evaluate the quality of clean reads. Then, high-quality clean reads were aligned to the reference genome of rainbow trout using BWA-MEM (v. 0.7.17) software. SAMtools (v. 1.9) software was used to extract these unmapped reads, including paired-end reads in which both ends are unmapped and unmapped single-end reads. De novo assembly was performed using the SOAPDenovo2 software with different K-mer sizes ranging from 81 to 127. The assembled contigs with length ≥500 bp were considered and treated with redundant deletion using CD-HIT (v. 4.8.1) software. Redundant contigs were removed with the identity of >95%. Quast (v. 5.2.0) software worked to evaluate the quality of several assembly versions constructed by the diverse K-mers. To avoid genomic contamination, a rigorous contamination filtering pipeline was performed for the contigs assembly by these unmapped reads. First, these contigs were aligned against the NT database using BLAST (v. 2.16.0) software and check the sequence homology of contigs. Then, the contigs were conducted with taxonomic classification by the Kraken2 software based on the public Kraken2-microbial database. It would help to ensure all these unmapped reads and contigs from rainbow trout. Finally, the contamination-free contigs were merged with the reference genome, generating the iterative pan-genome of rainbow trout.

### 5.3. Repetitive element annotation and gene structure prediction

Both de novo and homologue-based methods were applied for the annotation of repetitive elements in the assembled contigs and reference genome. First, de novo repetitive elements were identified and defined using the traditional pipeline of RepeatModeler (v. 1.0.7) software with default settings. The known repetitive elements of salmonids, including rainbow trout, Atlantic salmon and chinook salmon, were then extracted from the public Repbase (v. 20181026) database. Based on the references of both de novo and known repetitive elements, the prediction and categorization of repetitive elements were operated using the RepeatMasker (v. 4.1.3) software.

The structures of protein-coding genes in the repeat-masked assembly contigs were predicted using three different approaches, including RNA-based prediction, homology-based prediction and *ab initio* prediction. RNA evidence was derived from the 128 RNA-seq datasets of multiple tissues in rainbow trout, such as brain, gill, heart, liver, muscle and kidney tissues. These RNA-seq datasets were generated by our research group or published in the SRA database with the Bioproject ID of PRJNA638521 (96), PRJEB37848 (14) and SAMN29005439-SAMN29005456 (18). The clean reads from RNA-seq datasets were used for both de novo and genome-guided transcript assembly. Trinity (v 1.3.4) software was performed for the de novo transcript assembly. The bioinformatic pipeline of genome-guided transcript assembly was briefly as follows: clean reads were aligned to these assembled contigs using HISAT2 (v. 2.2.1) software. Based on the alignment, genome-guided transcript assembly was conducted using StringTie (v. 1.3.3) software. Then, the transcripts derived from both de novo and genome-guided assembly were merged and treated with redundant removing using CD-HIT (v. 4.8.1) software. It generated a complete set of the non-redundant transcripts of rainbow trout that acted as RNA evidence for following prediction of protein-coding gene structure. Homologous proteins were acquired from the three salmonids, namely Atlantic salmon, brown trout and chinook salmon. *Ab initio* prediction was largely depended on the accurate gene model. BRAKER2 (v. 2.1.6) software provided a fully automated training pipeline for the construction of highly reliable gene model of rainbow trout based on the available RNA-seq datasets mentioned above. Finally, structures of protein-coding genes were defined by integrating the RNA-based, homology-based and *ab initio* predictions with the MAKER (v. 3.1.4) pipeline. High-confidence gene structures were further filtered by the strict thresholds of annotation edit distance (AED) ≤0.5. The functional annotation of protein-coding genes was achieved by eggNOG-mapper (v. 5.0) software. The annotation completeness was assessed by BUSCO (v. 5.3.2) with the actinopterygii_odb10 database and the enrichment analyses of gene lists were achieved using the online DAVID tool (https://david.ncifcrf.gov/tools.jsp).

### 5.4. Presence/absence variations calling and gene content analysis

The clean reads from each accession were aligned once again to the pan-genome sequences of rainbow trout using BWA-MEM (v. 0.7.17) with default parameters, and the sequence depth of samples was calculated using Mosdepth (v. 0.3.3) software. PAVs and gene content were identified based on the cumulative coverage. The coding sequences (CDS) were extracted from the reference genome annotation file (GFF/GTF format). A custom Python script was used to calculate CDS coverage. The script utilizes BEDtools (v. 2.28.0) to generate per-base coverage depth for each CDS region. The coverage depth is then normalized by the total number of mapped reads to account for differences in sequencing depth across samples. Genes with CDS coverage ≥0.95 were considered present in the individual. Otherwise, it was defined as absent. Core and variable genes were defined based on the presence frequency in the 268 a9569896ccessions. Core genes were present in all individuals and variable genes were present in particular individuals. More specially, variable genes were majorly divided into three subcategories, including softcore, shell and cloud genes. In the present study, the frequency of core, softcore, shell and cloud genes were 100, 97–100%, 1–97% and less than 1% of the 268 accessions, respectively.

### 5.5. Presence/absence variation-based genetic structure analysis

The genetic information of PAVs was recorded and formed the VCF format by an in-house perl script. Then, it was converted to PLINK format (ped/map files) using VCFtools (v. 0.1.16). PCA based on PAVs was performed with GCTA (v. 1.94.1) software. The distance matrix was calculated using VCF2Dis (v. 1.45) and the maximum-likelihood phylogenetic tree was constructed based on the binary PAVs with using IQ-TREE (v. 2.2.2.6) software. The distribution of gene contents in rainbow trout from wild and selection populations was plotted using the pheatmap (v. 2.8.2) R package.

### 5.6. Presence/absence variation-based genome-wide association study and presence/absence variation-based fixation index analysis

GWAS and *F*_*ST*_ analysis were constructed to identify and characterize the PAVs associated with artificial selection. PAVs were filtered based on the strict criteria of MAF >0.05 and missing data <15%. In the genotype maps for GWAS (HapMap genotype file), the absent genes were represented by ‘A’, while the present genes were indicated as ‘G’. Common models for association analysis include general linear model (GLM), mixed linear model (MLM), multi-locus mixed linear model (MMLM) and fixed and random model circulating probability unification (FarmCPU). Using a Q–Q plot to consider the false positive in trait–marker associations and determine the best model. GWAS was conducted in GAPIT (v. 3.4.0) R package using the different model approach with the PCA matrix as covariate and kinship matrix as cofactor. The threshold for a significant association (*p*-value) was set based on the Bonferroni correction. The significant cut-off was defined as the threshold of –log10 (*p*) <5. Manhattan plots were produced using the CMplot (v. 4.5.1) R package. Meanwhile, high-confidence PAVs were used to calculate the frequency divergence of gene contents between wild and selection populations of rainbow trout using VCFtools software. The empirical threshold of top 1% *F*_*ST*_ values was considered for the significance.

## 6. Limitations of the study

To our knowledge, this is one of the first studies constructing the pan-genome and investigating the gene PAVs in distinct rainbow trout individuals with large amounts of sequencing data. However, there are still several limitations to the present study that we wish to address in the future. First, a series of protein-coding genes with abundant expression levels have been successfully predicted and annotated in the novel sequences. However, it fails to determine their corresponding physical locations on the chromosomes based on the current analysis methods, because of the novel sequences de novo assembled by the unmapped short reads. A considerable number of long reads should be further performed to fill these unknown gap regions and explore the complexity of rainbow trout genome among individuals. Another limitation was not systematically investigating the cellular and molecular basis regarding the important candidates of gene PAV linked to economic traits in rainbow trout. Therefore, additional studies *in vitro* and *in vivo* would be required to definitively determine the phenotypic effects of gene PAVs on growth performance and stress response in the future.

## Data Availability

Data: The data of selection PAV-based GWAS and selection signal analysis has been provided in the electronic supplementary material [82]. Pan-genome and WGS datasets can be provided upon reasonable request. Code: The in-house perl scripts used in this article can be provided upon reasonable request. All other requests: Any additional information required to re-analyse the data reported will be shared by the lead contact upon request.
